# The Organization of Foraging in the Fire Ant, *Solenopsis invicta*

**DOI:** 10.1673/031.011.0126

**Published:** 2011-03-08

**Authors:** Walter R. Tschinkel

**Affiliations:** Department of Biological Science, Florida State University, Tallahassee, FL 32306-4370

**Keywords:** colony census, colony growth, division of labor, forager density, foraging tunnels, labor allocation, ortstreue, route faithfulness, seasonality, superorganism, territory, trail recruitment, worker age

## Abstract

Although natural selection in ants acts most strongly at the colony, or superorganismal level, foraging patterns have rarely been studied at that level, focusing instead on the behavior of individual foragers or groups of foragers. The experiments and observations in this paper reveal in broad strokes how colonies of the fire ant, *Solenopsis invicta* Buren (Hymenoptera: Formicidae), allocate their available labor to foraging, how they disperse that force within their territory, and how this force changes with colony size, season and worker age. Territory area is positively related to colony size and the number of foragers, more so during the spring than fall. Changes of colony size and territory area are driven by seasonal variation of sexual and worker production, which in turn drive seasonal variation of worker age-distribution. During spring sexual production, colonies shrink because worker production falls below replacement. This loss is proportional to colony size, causing forager density in the spring to be negatively related to colony and territory size. In the fall, colonies emphasize worker production, bringing colony size back up. However, because smaller colonies curtailed spring worker production less than larger ones, their fall forager populations are proportionally greater, causing them to gain territory at the expense of large colonies. Much variation of territory area remains unexplained and can probably be attributed to pressure from neighboring colonies. Boundaries between territories are characterized by “no ants' zones” mostly devoid of fire ants. The forager population can be divided into a younger group of recruitable workers that wait for scouts to activate them to help retrieve large food finds. About one-third of the recruits wait near openings in the foraging tunnels that underlie the entire territory, while two-thirds wait in the nest. Recruitment to food is initially very rapid and local from the foraging tunnels, while sustained recruitment gradually involves the recruits waiting in the nest. As recruits age, they become scouts searching for food on the surface, and die about two weeks later. Foraging tunnels decrease in cross-sectional area with distance from the nest, in keeping with the gradual bleeding off of workers to the surface with distance. Foragers lack route-faithfulness, and having been marked and released at one point within the territory, they can be recaptured at any other point a day later. The size of the territory actually occupied may be limited during dry weather, resulting in very large no-ants' zones.

## Introduction

The behavior of individual foraging ants is a consistent element in natural history studies of most ant species. Using the search term “foraging” in the ant literature database Formis (Wojcik et al. 2008) retrieves almost 3,000 papers, about 10% of all papers published on ants. Restricting the occurrence of the search term to the title or keywords still nets over 2,700 papers, and eliminating conference abstracts, 2,000. Topics range from food choice to recruitment behavior to competition and so on, but very few of these papers deal with foraging from the superorganism point of view in which the ant *colony* is regarded as a single functional entity that allocates time and resources to competing internal functions, be they brood care, alate production, foraging or others. From the point of view of evolution, it is at this level that natural selection acts most strongly, resulting in some (presumably) optimal allocation pattern. Individual, or even groups of workers and their responses are simply the internal machinery of a colony-level phenomenon evolved (perhaps) to optimize the return on a given level and pattern of allocation.

To illustrate with an analogy, the study of foraging has been as if the study of an organism (e.g. a mammal) had focused almost exclusively on the workings of liver cells, without considering how the liver as an entire organ contributes to the organism as a whole. Undoubtedly, many aspects of foraging, such as trail recruitment, searching behavior, selforganization, food choice, travel distance, competition and so on are interesting subjects in their own right, but from the superorganism point of view, the questions should more properly be how much worker biomass, energy and time does the colony devote to foraging; how does it spread this allocation in time and space, what return does it get from this allocation, and how do these patterns change during the life cycle and seasons? Foraging strategies, while more easily studied on the basis of individuals or groups of workers, evolve through natural selection on colonies and exert their important effects at the colony level. In contrast to the intense scrutiny that the easily observed and quantified elements of foraging strategies have received, superorganismal foraging strategies of ants have seldom been investigated. This is not to say that elements of such strategies have received no attention at all, it is that they have not been clearly interpreted as adaptations of the superorganism.

Because ants live in fixed colonies and emanate from them to forage, they are referred to as central place foragers, returning any food to the colony (Hölldobler and Wilson, 1990). This pattern is less obvious when the ant colony is polydomous, with workers moving among multiple scattered nests. Nevertheless, even in such cases, the ants forage locally and are mostly faithful to a particular colony subunit, creating a system best described as dispersed central place foraging (McIver and Loomis 1993; Buczkowski and Bennet 2006; Heller et al. 2008). This divides the population into smaller clusters of multiple nests that show little exchange with neighboring clusters. Faithfulness to a home nest and little exchange with neighbors also characterizes the polygyne social form of the fire ant, *Solenopsis invicta* Buren (Hymenoptera: Formicidae) (Ross 1993), suggesting that it too is a dispersed central place forager.

Studies of foraging by ants have been dominated and guided largely by optimal foraging theory (Schoener 1971; adapted to social insects by Oster and Wilson 1978). Many have tested the various hypotheses of this theory, in particular, whether individual ants behave in a manner that maximizes their net energy gain from foraging (e.g. Bailey and Polis 1987; Bonser et al. 1998; Clark 1994; Detrain et al. 2000: Pyke et al. 1977; Reyes López 1987; Rockwood and Hubbell 1987; reviewed in Hölldobler and Wilson 1990). A few have asked whether there is a connection between optimal individual strategies and optimal colony strategies or have investigated the collective effect of forager actions (for example: Bernstein 1975; Beekman et al. 2001; Adler and Gordon 2003; Goss et al. 1989; Kay 2002; Cole et al. 2008; Burd 2000). In the current paper, I am less concerned with such finer points of worker decision-making and more concerned with whole-colony patterns in broad strokes.

A major impediment to studying foraging at the whole-colony level is that even when estimating the number of foragers, investigators usually do not census the entire colony (e.g. Tripp et al. 2000; Cole et al. 2008) and thus cannot judge the relative allocation of labor to foraging or other functions, nor can they estimate the foraging intake in relationship to colony size and needs. In the few cases that included a colony census, the forager population was usually a small proportion of the total worker population. Kruk-DeBruin et al. (1977), Ayre (1962), Chew (1959), Golley and Gentry (1964), Goss et al. (1989) and Porter and Jorgenson (1981) marked ants collected from the surface (not from within the nest) and found that their mark-recapture estimates were only a minority of the real nest population. In *Formica polyctena* weeks-long marking of workers captured on trails failed to mark a large percentage of the colony, showing instead that the forager population consisted of a separate group of individuals (Kruk-DeBruin 1977). Together, these papers showed that the forager population was a distinct, only partly-overlapping, small subset of the entire colony. In contrast, most of the colony of *Pachycondyla caffraria* forages (Agbogda and Howse 1992). Unfortunately, the data are too scarce to detect whether there are any phylogenetic or colony size effects on the allocation of labor to foraging.

When it is economically and energetically profitable, ant colonies may compete for resources by defending a territory. Such territories can be absolute, that is, defended all of the time, or spatio-temporal when only crucial resources are defended, and these resources vary in time or space (Hölldobler and Wilson 1990). Even in the absence of active territory defense, foraging areas may not overlap because the ants have behavioral means of avoiding contact with neighbors (Bernstein 1975; Harrison and Gentry 1981). The fire ant, *S. invicta*, defends an absolute, sharply-bounded territory against its neighbors. The size of this territory is proportional to the colony size in the spring (Tschinkel et al. 1995), but also depends upon the size and density of competing fire ant colonies within its neighborhood (Adams 2003).

The fire ant, *S. invicta*, is well-suited for the investigation of colony-level foraging effort and patterns. Each colony defends a territory within which it forages and from which it excludes all neighboring workers, so that within each territory, the colony-origin of foragers is unambiguous. While foragers may return small items without recruiting nestmates, *S. invicta* is known for its robust recruitment to large food items. Colonies grow through five orders of magnitude, allowing ready testing of size-related patterns of allocation of workers to the foraging force. Finally, the large literature on the biology of this exotic ant has been summarized by Tschinkel (2006).

This paper summarizes what I have learned about monogyne fire ant foraging over more than 15 years of experimentation and observation on the patterns of allocation of workers to foraging, the association of these patterns with worker demography, the creation of foraging territories and the dispersal of foragers within them.

## Materials and Methods

### Study sites

All study sites were in the vicinity of Tallahassee, Florida, in pastures or lawns. Between 1996 and 2005, studies were carried out in three different improved pastures at Southwood Plantation. After this time, studies were sited at the Miccosukee Greenway northeast of Tallahassee, and after 2007 at Innovation Park in Tallahassee. All colonies in this study were of the monogyne social form.

### Mound volume

Colony size was estimated from the mound volume (Tschinkel 1993).

### Determination of colony territorial boundaries

**Figure 1.**
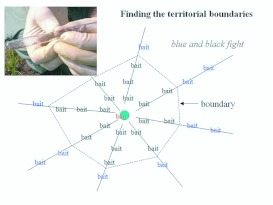
Layout of bait tubes for determining territorial boundaries. Once ants have recruited to the baits, tubes are brought mouth-to-mouth (inset), beginning at the mound and working outward. Fighting workers indicate that the workers come from different nests, and that the boundary lies between these last two tubes. High quality figures are available online.

When workers from different monogyne *S*. *invicta* colonies are brought into contact, they often fight. This hostility has been used to determine the boundaries of the territory exclusively occupied by each colony (Tschinkel et al. 1995) (workers from different polygyne colonies do not fight with each other and do not defend territories). Small test tubes with small pieces of Spam or tuna were laid on the ground at 1 to 2 m intervals on spokes radiating outward from the focal colony at the center (Figure 1). Several bait tubes were also placed on a board on the colony mound itself. All these baits were typically found by the ants within less than 15 min. On each radius, workers in one of the more central test tubes were brought into mouth-to-mouth contact with tubes from the mound. If no fighting was observed within approximately 2 minutes, workers in the test tubes were judged to be from the focal colony. These test tubes were then placed mouth to mouth with the next outward tube on that radius, and the process repeated until fighting was observed. The boundary was located between these last two test tubes. Its location was sometimes determined with more precision by adding bait tubes between these two.

The boundary points on 8 to 12 such radii were mapped using polar coordinates with the colony at the origin. The polygon that resulted from connecting these points is a map of the territory. All further procedures were carried out within the territory of each focal colony, so that any captured foragers unambiguously originated from that colony.

### Marking and recapturing foragers

Up to 20,000 foragers were captured at ample baits on small boards scattered throughout the territory of the focal colony. These were combined and weighed as a group on a balance in the field, and a sample of about 100 was set aside for later determination of the mean weight of individual foragers. The foragers were then lightly anaesthetized with ether, scattered thinly in the bottom of a tray and sprayed lightly with 3% fluorescent printers' ink (Day-Glo orange, or fluorescent yellow) in diethyl ether. The marked ants did not appear conspicuously colored to the naked eye, but illumination with ultraviolet light revealed nearly 100% of them to be marked. Once the marked, anaesthetized ants were fully active again, they were released either in small clumps at random points throughout the territory or at specified locations within the territory, depending on the experiment. These procedures were usually completed before noon, as foraging was greatly reduced by midday heat. Colonies were used only once. Marked ants seemed to behave normally and not rejected by their nestmates.

Foragers were recaptured by placing small test tubes with tuna or Spam throughout the territory and plugging the tubes with cotton after they had been occupied by abundant recruits. These were killed in the laboratory by freezing, and the numbers of unmarked and fluorescent-marked ants were counted.

The number of marked ants released, together with the proportion of the recaptured foragers that were marked, allowed estimation of the number of recruitable foragers. Only data from colonies with more than an 8% recapture rate were used. Recapture rates below 8% gave highly variable estimates.

### Estimation of the nest population

Estimation of the recruitable population as a fraction of the total requires that the focal colony's nest be censused as well. This was done two days after the forager estimation by excavating the colony (mound and below-mound portions separately to check for stratification of foragers in the nest) into bins, weighing the total amount of dirt and ants, mixing the ants and dirt homogeneously, and removing 5 approximately 200 g samples from this mixture (Tschinkel 1993). A count of the various ants in these samples allowed the estimation of the total number in the nest as the number in the sample divided by the fraction of the total weight that the sample represented. The mean of all samples was used as the estimate of the worker populations in the mound and below-mound portions, and their sum was the total nest worker population. Adding the nest population and the recruitable population gave the total colony worker population. Census of the focal nest took place two days after marking workers. The mean dry weight of workers was determined in the laboratory for both forager and nest samples.

Fluorescent-marked workers also appeared among the nest workers. These were probably foragers that happened to be in the nest at the time it was excavated. Their numbers were calculated from knowledge of the proportion of recaptured recruits that were marked, and added to the recruitable population. Subtracting them from the nest population gave an estimate of the non-recruitable nest population. In this way, the total colony population was partitioned into two major categories — nest workers and recruitable, forager workers. Because the territory area had been determined, the forager density within the territory could be calculated.

These procedures were carried out on colonies of a range of sizes in the spring of 1996 and again in the fall. I did this because *S. invicta* colonies are largest in mid-winter and smallest in mid-summer after producing sexuals (Tschinkel 1993). It seemed possible that this seasonal cycle would also affect the characteristics of the foraging force (and it did!).

### Direct collection of foragers by vacuum

In the spring of 2006, I determined the scout density directly by collecting foraging fire ants using a portable, battery-powered vacuum cleaner (DeWalt, 18 v, www.dewalt.com).

The territorial boundaries were determined as described above, and the boundaries marked with flags and spray paint. Two days later, a metal square enclosing 0.1 m^2^ was placed at several successive locations within each territory. All litter within the square was scraped up by hand, and the ground and grass within the square thoroughly vacuumed. The litter and material in the vacuum container were combined and bagged. Such samples were more or less evenly spaced along 3 to 8 radii from the focal colony to the territorial boundary (and in some cases, beyond). A total of ten colonies, 5 large and 5 small, were sampled in this manner. All sampling was completed in the morning before 11:30 a.m. or before soil surface temperatures exceeded 35° C.

The bagged material was searched for fire ants by scattering small amounts of it at a time in a white tray. Ants immediately scampered and were collected by aspirator for later measurement of headwidth. The effectiveness of this sampling method was tested by vacuuming a second sample immediately after the first. These second samples rarely contained ants, and when they did, these comprised a small percentage of the first sample.

### Casting foraging tunnels

Zinc was melted in a stainless steel ladle over a propane burner surrounded by insulation. Foraging tunnels were exposed by trenching around the nest, and the openings were supplied with a sprue made from damp soil. Molten zinc was poured into the sprue until draining stopped. Casting always proceeded outward from the nest. The frozen cast section was exposed by removing the overlying soil but was left in place for later photography and labeling.

### Data Analysis

Data were log-log transformed as needed to stabilize the variance and analyzed by multiple regression, using dummy variables for season. The best model was selected by removing variables without significant effect one at a time.

## Results and Discussion

### Colony size, foragers and territory area

The mark-recapture study produced a large set of interrelated variables to be sorted into a plausible sequence of relationships. The basic variables of mound volume, nest census, forager estimates, territory area, mean worker or brood dry weight or fat content, were combined to calculate such variables as total colony weight, total colony worker population, forager density, area per worker and area per forager.

In order to make sense of these relationships and to limit the number of comparisons, I have reasoned as follows. The measures of colony size - total number of workers or total colony weight - are probably the basic drivers of most of the other variables. It was assumed that factors associated with colony size drive forager population, which in turn drives territory area, though there is clearly also a feedback loop from both back to colony size. Moreover, in fire ants, territory area is not dependent on the focal colony alone but is the outcome of neighborhood interactions (Adams 2003). Increased colony size also increases both worker size and worker fat content (Tschinkel 1993), possibly through improved nutrition. Worker size may also directly affect the territory area per worker. Differences in the rates with which these variables increase with colony size or forager population result in shifting ratios — such as worker density or area per worker — that can be either positively or negatively related to any measure of colony size.

Because territory is defended by worker behavior, it is likely to be more strongly associated with the number rather than weight of ants. Therefore the total worker population (hereafter called “colony size”), rather than total colony weight (hereafter, “colony weight”) was usually used as the measure of colony size. Territory can be thought of in two different contexts — as the source of food and shelter for the colony and as the outcome of worker territorial behavior. In a uniform environment, the food yielded by a territory would, on average, increase at the same rate as territory size. Therefore, one would expect territory area to keep pace with the increase in colony biomass, because biomass is the source of the demand for food (demand may actually increase somewhat less because as colony and worker size increase, metabolic rate decreases). This expectation is borne out in the spring sample: the relationship of colony weight to territory area is isometric, as it was in Tschinkel et al. (1995). The slope of the log-log plot was 1.16, not significantly different than 1.0. Territory area thus keeps pace, or is slightly ahead, of the demand for food. During the fall, colonies produce large numbers of workers, and territory area lags far behind colony weight, a relationship also seen in the number of workers vs. territory area (below).

**Figure 2.**
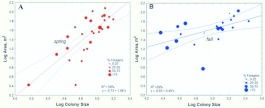
The territory area in relationship to the number of workers in the colony for the spring sample (A) and the fall sample (B). Bands are 95% Cl for the regressions. The size of symbols codes for the percent of the colony that forages. The slopes differ significantly between seasons. Regression statistics can be found in Table 1. High quality figures are available online.

The interpretation of the regressions is greatly complicated by seasonal differences, beginning with the relationship between colony size and territory area (Figure 2; Table 1). As background, it is necessary to understand that sexual-producing fire ant colonies undergo large seasonal changes of colony size (about 2-fold) because while the colony is producing sexuals in the spring, worker production is reduced below replacement, and the colony gets smaller by as much as half or more. In late summer, after sexual production has more or less ceased, the colony produces mostly workers, causing the colony to double or more in size, compared to its summer minimum (Tschinkel 1993; 2006). These size changes take place within the limiting constraints of the colony's territory, because all colonies are hemmed in by their neighbors, and changes in the size of one territory can occur only through changes in those of neighbors (Tschinkel et al. 1995). This loss or gain of territory depends, of course, on the nature of a colony's neighbors, but even so it is not entirely random. Over the annual cycle, large territories tend to lose area, and small ones to gain it at the expense of large ones (Adams 2003), creating a ratchet like mechanism that allows small colonies to move up to reproductive size while large colonies lose size during alate production (Tschinkel 2006). The reason for this is that smaller colonies invest more heavily in workers and less in sexuals, allowing them to gain territory at the expense of large ones and adding great complexity to the interpretation.

In the spring, territory area is strongly related to colony size (Figure 2A). A 10-fold increase in colony size is associated with a 30-fold increase in territory area. In sharp contrast, fall territory area is only weakly associated with colony size (Figure 2B), and a 10-fold increase in colony size is associated with only a 3-fold increase in area. For large colony sizes, territories fall in the same size range in both seasons, but in the fall, small colonies of a given size command a much larger territory than comparable-sized colonies in the spring. Most likely, this is space they have won at the expense of larger colonies that were busy producing sexuals.

**Table 1.**
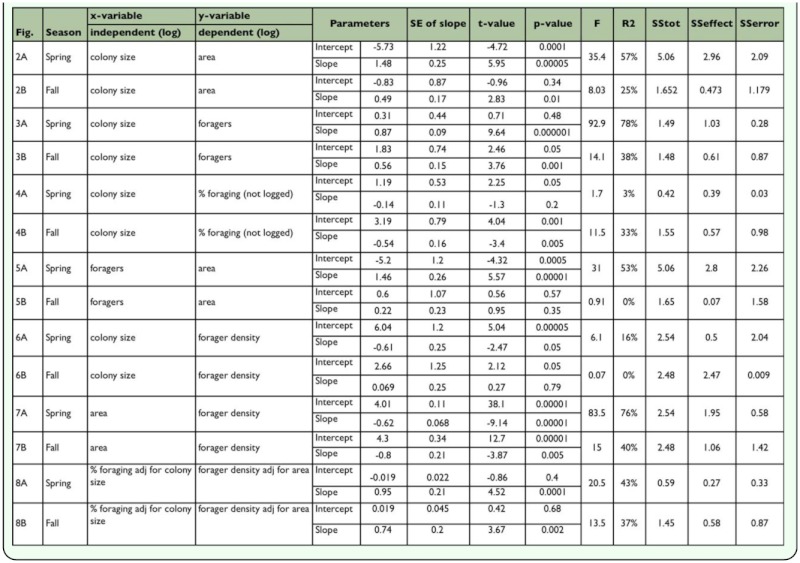
Regresssion analyses of various estimates of colony size and forager populations. All spring regressions had 1, 25 degrees of freedom, and all fall regressions 1, 20.

**Figure 3.**
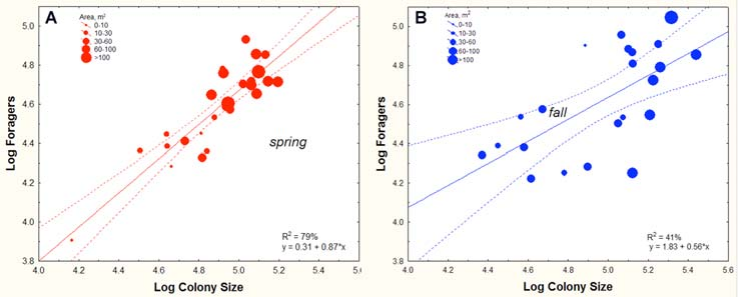
In both the spring and the fall, the population of foragers increased more slowly than the worker population as a whole. However, the slope is significantly different from 1.0 (isometry) in the spring at only the 0.1 level, but is much less than 1.0 in the fall. In the fall, a 10-fold increase in colony size is accompanied by 3.6-fold increase in foragers. Averaged over the two samples, a 10-fold increase is associated with an approximately 5-fold increase in foragers. The size of the symbols codes for the territory area. Regression statistics can be found in Table 1. High quality figures are available online.

It seems likely that territorial interactions are limited to foragers, so the size of the territory should depend more strongly and directly on the population of foragers than on the colony population as a whole. Figure 3 shows the relationship between colony size and the number of foragers.

In the fall the forager population increased by only about 3.6-fold for every 10-fold increase in the number of workers (Figure 3; Table 1). In the spring, a 10-fold increase in colony size was accompanied by a 7.4-fold increase in foragers, but the slope was only different from 1.0 (isometry) at the 0.1 level (Figure 3A). Combining both samples showed that over the year foragers increased at about half the rate as colony size. These slopes of less than 1.0 indicate that the fraction of a colony that foraged decreased with colony size, and this relationship was much stronger and less variable in the fall (Figure 3B) than the spring.

**Figure 4.**
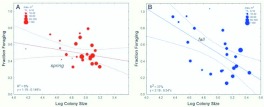
In the spring, the fraction foraging was not significantly related to the colony size, but in the fall, the fraction foraging declines by 60% for every 10-fold increase in colony size. The effect of colony size on area can be seen in the increasing size of the symbols (which are coded for territory area) at larger colony sizes. Regression statistics can be found in Table 1. High quality figures are available online.

This was confirmed by plotting colony size against the fraction foraging (Figure 4; Table 1). In the spring, the fraction that foraged was not significantly related to colony size (Figure 4A), but in the fall, every 10-fold increase in colony size was accompanied by an approximately 60% decrease in the fraction foraging (Figure 4B). Moreover, the range of this fraction was greater in the fall (approximately 10–90%) than in the spring (30–80%). Smaller fall colonies fielded a much higher fraction of foragers than their spring counterparts and larger fall colonies a lower fraction. A likely source of these differences is the different age-structure for small and large colonies in the fall. By the fall, large colonies have probably lost a large proportion of their older workers, the foragers, because they reduced the production of new workers in inverse proportion to sexual production during spring. As they aged, this reduced worker population supplied the foragers in the fall. Smaller colonies, on the other hand, curtailed worker production less or not at all, so that by fall, the proportion of older workers (foragers) was greater. These differences in small and large colonies produced the pattern seen in Figure 4B. During the winter, worker production in colonies of all sizes is greatly reduced, so that by the time sexual production begins in spring, the proportion of old workers (foragers) is no longer related to colony size (Figure 4A), averaging about 50%.

**Figure 5.**
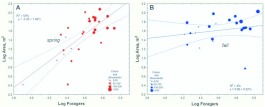
the spring, a 10-fold increase in the population of foragers is associated with a 30-fold increase in territory area, suggesting that the area held per forager is much greater in large than in small colonies. In the fall, there is no relationship between the forager population and the territory size. Regression statistics can be found in Table 1. High quality figures are available online.

**Figure 6.**
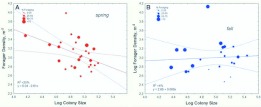
Forager density is negatively related to colony size in the spring, but independent of it in the fall. This is probably caused by reverse processes in the two seasons, a loss of foragers in the spring that is more than proportional to colony size, and a production of new foragers in the fall that is inversely proportional to colony size. Regression statistics can be found in Table 1. High quality figures are available online.

Figures. 3 and 4 show that the forager population is not isometric with colony size. Ultimately, foragers (or some fraction of the forager population) must be responsible for gaining and holding territory, so the relationship of the forager population to the area of the territory is of central interest, and is shown in Figures 5 and 6 (regression statistics in Table 1). Again, the situation is very different in the spring and fall (Figure 5A, 5B). In the spring, a 10-fold increase in the forager population is associated with a 30fold increase in territory. In large colonies, each forager occupies much more territory than she does in small colonies. This situation could arise in at least two sharply divergent ways. First, foragers in larger colonies could actually become very much more effective in gaining and holding territory wrested from their neighbors. However, a second way is more likely — colonies could lose foragers during the spring (as noted above), and this loss could be proportionally greater for larger colonies. Moreover, although this loss of foragers may be associated with some loss of territory, the loss is less than proportional, so that larger colonies end up with lower forager densities than smaller ones (Figure 6A). In other words, the situation arises mostly through forager *loss* rather than territory *gain*. Whereas large territories do lose more area to small ones over the annual cycle (Adams 2003), the magnitude of these losses cannot explain a 30-fold increase in area per-forager for every 10-fold increase in foragers (Figure 5A). This less than “expected” loss of territory probably results from the buffering of territory loss against modest losses of foragers (Adams 2003). Changes in territory size are never rapid (pers. observations) under natural circumstances. The outcome of these seasonal changes in the forager populations is that seen in Figure 6 — a negative relationship between colony size and forager density in the spring, and no relationship in the fall.

**Figure 7.**
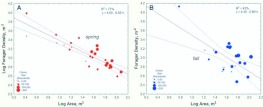
Variation around the mean regression of area on foragers is shown here as the foragers per m^2^ in relation to area. For a given number of foragers in Figure 5, the density of foragers necessarily drops as area increases. This effect is largely, but not entirely, responsible for the relationships in this figure. Regression statistics can be found in Table 1. High quality figures are available online.

Figure 7 is best understood as showing the variance around the mean regression of area on foragers — the measure foragers per m^2^ removes the average effect of territory size itself. A slope of zero would indicate that the density of foragers does not change with area, but Figure 7A, 7B (regression statistics in Table 1) shows that forager density decreased with area in both spring and fall. Colony size made a modest contribution to this variation in the spring (Figure 6A; size-coded symbols in Figure 7A), but not in the fall (Figure 6B; size-coded symbols in Figure 7B). This large area variance is associated with other factors, most likely territorial pressure within neighborhoods. A single point gives the fall regression (Figure 7B) a high R^2^ value of 43%; its removal drops the R^2^ value to 10% (n.s.) and the slope to -0.36. Figure 7 also shows that territory area and density are less variable in the fall. Thus, in the spring, as the area increased by 10-fold, the forager density decreased by 4-fold (i.e. to 25%), but in the fall, this relationship is of questionable significance.

Although colonies holding larger territories generally had a larger colony size (coded by symbol size in Figure 5), this relationship was not tied exactly to the forager population because colonies differ in the proportion of workers that forage (Figure 4). Colony size was unrelated to percent foraging in the spring, but strongly and negatively related in the fall. However, in both seasons there was a very large variation around the relationship of area to colony size (Figure 2), suggesting the question, Do colonies that field a higher proportion of their work force as foragers gain territorial advantage, or does the addition merely increase the forager density by the same proportion? That is, does the forager density increase more slowly than the percent foraging?

**Figure 8.**
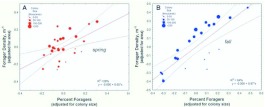
After adjusting the area per forager for territory area, and percent foraging for colony size, this plot reveals that fielding a higher percentage of foragers in the spring is associated with a net decrease of forager density (slope = -0.65). But in the fall this association is not present (slope= -1.0). Regression statistics can be found in Table 1. High quality figures are available online.

To test this, forager density (adjusted for territory area) was plotted against the percent foraging (adjusted for colony size) (Figure 8; Table 1) (i.e. the residuals from two regressions). This removes the effects of area on density and colony size on % foraging, leaving the variation that is not explained by these two factors. Again, the results are very different by season. If fielding a higher percentage of foragers gained no additional territory, merely adding in exact proportion to the forager density, the slope in Figure 8 would be 1.0. In the spring, a 10-fold increase in the percent foraging (beyond that associated with colony size) was associated with an approximately 5-fold increase in area-adjusted forager density (Figure 8A; the slope 0.65 is smaller than 1.0 at p= 0.062; t_25_ = -1.72). In other words, there was a net gain of territory per worker, or alternatively, colonies that fielded a higher proportion of foragers also sustained a higher-than-proportional loss of foragers during the spring. In the fall, a 10-fold increase in percent foraging (beyond that associated with colony size) increased the density exactly 10-fold, that is, added in exact proportion to the density without any territorial increase (Figure 8B). The remaining variation around the regressions in Figure 7 is due to factors not measured in this study, most likely deriving from neighboring colonies. Colonies find themselves in different neighborhoods, and thus experience great variation in the “territorial pressure” exerted by their neighbors. The variation around the regression line would thus represent different degrees of compression by neighbors. The original mark-recapture study did not estimate the size and density of neighbors and thus cannot test neighborhood effects.

To sum up, the processes that probably created these patterns are the seasonal changes of colony size and worker demography (agedistributions). During the annual cycle, changes in colony size are positively related to their capacity to produce sexuals. The proportional reduction of worker production during sexual production (Tschinkel 1993; 2006) creates an annual cycle of worker age distribution that differs by colony size. Large, sexual-producing colonies, in addition to losing more colony size, become disproportionally depleted of older workers. Because older workers make up the forager class (see below; Mirenda and Vinson 1981), and foragers defend the territory, their forager density declines, and they lose territory to smaller colonies. Smaller colonies producing few or no sexuals produce proportionally much larger numbers of workers during the spring, boosting their colony size, but also pushing increased numbers of aging workers into the field as foragers early in the fall, thus boosting forager density relative to large colonies in the fall. As a result, these smaller colonies gain territory at the expense of the larger, sexual-producing ones, by moving the low end of the curve upward (Figure 2 B) by rotating the mean regression line around approximately 100,000.

### Direct estimation of forager density

**Figure 9.**
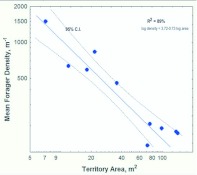
Forager density as estimated by vacuum sampling in relationship to territory area. Forager density decreased by 85% over the range of territory areas. High quality figures are available online

The number of foragers captured by vacuum sampling per 0.1 m^2^ ranged from 0 to almost 300. Averaging samples within territories showed that, as for the mark-recapture study, forager density depended strongly on the size of the colony and its territory (Figure 9). Small colonies averaged between 800 and 1500 foragers per m^2^, whereas the largest colonies averaged only about 250. In two colonies, forager density was highest within 1 m of the mound and decreased outward, but this pattern was not detectable in other colonies.

The structure of the mown lawn consisted of clumps of grass, the ground surface between clumps typically covered with a layer of grass-clippings in various stages of decay. How might the forager population be vertically distributed in this situation? To determine this, the grass and surface of the litter layer was vacuumed, then the litter was scraped together, and then the exposed ground surface was vacuumed. These three samples were bagged and analyzed separately.

By far the largest number of foragers occurred in the ground vacuum samples. Three vacuum samples from each of two colonies showed a mean of 9.7 (SD 4.1) workers in the top sample, 13.3 (SD 8.3) in the middle and 77 (SD 8.0) in the ground layer sample. These numbers indicate that the ants were probably mostly on the ground surface, just below the litter. Indeed, careful lifting of the litter often revealed trail-like indentations in the ground, with foragers running in these grooves.

**Does forager density vary within the territory?** It seemed possible that forager density varied with distance from the nest, perhaps being higher near the nest or near the boundary. Multiple regression with dummy variables for colony showed that only one of the ten colonies showed a significant decrease in forager density from the nest to the boundary. However, this same colony was one of the smallest, with the smallest range of nest-to-boundary distances, and the highest forager densities. The significance of the slope disappeared in a simple distance-density regression for this colony. It seems unlikely that there is any meaningful relationship between the distance from the nest and the forager density.

It is also possible that the foragers are not evenly distributed within the territory. Histograms of the frequency distribution of foragers in vacuum samples showed a strong right skew for most colonies, with a small minority of samples containing several-fold the average number of foragers. Many of these were probably recruitment events and contributed to the uneven distribution of foragers among samples. Even distribution would result in the mean forager density at every sample site, so the observed densities were compared to this expected value using a χ^2^ test. All of the colonies showed highly uneven distribution of foragers among the individual vacuum samples. All p-values were less than 0.0000001. No doubt, recruitment events are responsible for some of this unevenness, but it also seems likely that the location of foraging tunnel exits would influence forager densities. Analysis of patterns to this level of detail must await future research.

**Figure 10.**
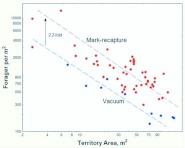
A comparison of forager densities estimated by vacuum sampling and by mark-recapture using baits. The slopes of density against area are identical, but the values of the mark-recapture estimate for any given territory area are always 2.2-fold greater, suggesting that only about 45% of the recruitable workers are scouts active on the ground surface. High quality figures are available online.

**Vacuum sampling vs. mark-recapture estimates:** Vacuum sampling estimates the population of foragers on the ground surface or vegetation, that is, the population of scouts searching for food, or recruits to larger food finds. It is likely that scouts greatly outnumber recruits in these samples as large food finds are probably not common. On the other hand, mark-recapture estimates the total population of foragers that can be recruited to food. Comparison of the estimates by these two methods gives insight into what proportion of the forager population consists of scouts and recruits, respectively. Figure 10 makes this comparison (because vacuum-sampled colonies were not censused, comparisons can only be made on the basis of territory size). Remarkably, the slope of density vs. territory area (both logged) is almost exactly identical between the two sampling methods — a doubling of territory area results in an approximately 50% decrease in density. However, densities measured by mark recapture averaged about 2.2 times larger than those by direct vacuum sampling, suggesting that only about 45% of the total forager population was available on the ground surface for vacuum sampling. The remainder must have been either underground or in the nest mound. This possibility is tested below. Hereafter, ants captured by vacuum are referred to as *scouts*, and those estimated by mark-recapture as *recruits* (although each group undoubtedly also contains some of the other).

**Do recruits become scouts as they age?** Recruits are brought forth from their underground waiting places, be these in the foraging tunnels or in the nest. Hypothetically, scouts differ in behavior from recruits, because scouts are active on the ground surface, while recruits wait underground. If scouts and recruits are really distinct groups, it should be possible to demonstrate this through mark-recapture experiments. Foragers that are captured on baits and marked should consist mostly of recruits. Workers recaptured on baits (recruits) the next day should have a higher proportion of marks than those recaptured in vacuum samples or aspirated from the ground (scouts). Moreover, if scouts become recruits as they age, as seems likely, the temporal rate of disappearance of marks among scouts should lag that of recruits.

This experiment was executed as follows. The territory boundaries of four colonies were mapped as before. On day zero, a dozen small boards festooned with strips of Spam were placed in each territory, and foragers coming to them were accumulated in trays with a fluon barrier. When between 16 and 35 g (16 to 35 thousand) of foragers had been collected, these were anaesthetized with ether and sprayed lightly with fluorescent printers ink (dayglo orange, or signal yellow) diluted 1:20 in ether. Upon waking, a small sample was withdrawn for determining worker weight and percent marked, and the remaining marked foragers were returned to their own territories.

On day one, and again on days 7, 14 and 21, foragers were recaptured in three different ways. Initially, “scouts” were captured by vacuuming six to eight 0.1 m^2^ samples within the territory, but later scout samples were collected by simply aspirating wandering workers on the ground surface within territories, taking care to avoid obvious recruitment events. Whereas vacuuming also collected whatever recruits happened to be in the sample area, direct aspiration probably avoided most such errors and represents a “purer” sample of actual scouts. Recruits were recaptured in small test tubes with bits of Spam inside. When a few hundred ants had accumulated inside them, the tubes were plugged with cotton. All samples were taken to the laboratory, frozen to kill the ants, and inspected carefully under a microscope using UV illumination in a darkened room. In the violet gloom of the UV light, even tiny spots of fluorescent ink shone like beacons.

This procedure was replicated on 5 different colonies that ranged in territory area from 26 to 80 m^2^, and one colony was run twice, using different colors (as expected, the estimated forager population was strongly and isometrically related to the territory area; loglog regression; F_1,4_ = 12.24; R^2^ = 69%)

**Figure 11.**
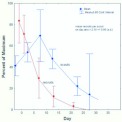
Foragers captured on baits (recruits) were marked and released on day 0. On day 1 through 29, workers were recaptured on baits (recruits) or wandering on the ground surface (scouts), and the proportion marked determined. The decline of marked scouts lags that of marked recruits suggesting that recruits are a different subpopulation from scouts, and become scouts as they age. The initial increase in marked scouts also supports this transition. Error bars are 95% confidence intervals. High quality figures are available online.

On day one, mark rates of recaptured ants ranged from 12 to 43%. Because absolute mark rates varied among replicates, all rates were standardized to a common scale as percent of the highest mark rate in each replicate (Figure 11). The day zero values were not directly measured, but were calculated as follows. Between day 1 and day 21, the mark-rate of the recruit group decreased about 15% per day (i.e. the decline was linear on a semilog plot). Thus, on day zero (the day on which the foragers were marked), there were 15% more than on day one. Correspondingly, this correction was subtracted from the scout value for day one to produce the scout mark-rate for day zero.

As predicted, initially a smaller proportion of the scouts than recruits were marked, indicating that workers recruited to baits are an only partially overlapping population with those that scout on the surface. Using the values for day zero, the mean recruits per scout was 2.16 (SD 0.95; 95%CI, 1.16–3.16). In other words, about one-third (32%) of the foragers were acting as scouts, a value somewhat lower than the estimate (45%) derived from vacuum samples. However, as noted previously, vacuum sampling also captures recruits that happen to be busy retrieving food, and therefore probably overestimates the number of scouts. The value derived from mark-recapture is probably more accurate.

By the end of the first week, the proportion of marked scouts had increased while that of recruits had fallen. This suggested that recruits become scouts, boosting their mark rates and decreasing the mark rates of the recruits. The mark rate of recruits declined exponentially (i.e. was linear on a semilog plot), indicating that a fixed percentage (about 15%) of the recruit population made the transition every day.

From week 2 onward, the mark rate of both scouts and recruits decreased until by week 4, there were no marked recruits, but a considerable fraction of scouts were still marked. These patterns are best explained as resulting from the transition of recruits to scouts, who die at roughly the same rate that recruits become new scouts. The spacing of the two curves on the horizontal axis suggests that scouts live about 2 weeks.

**Figure 12.**
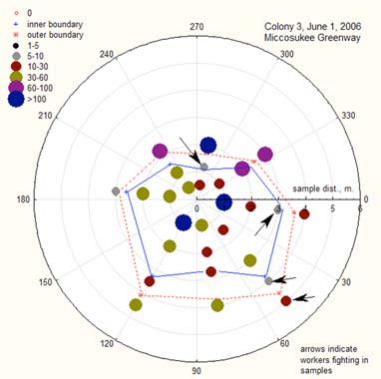
Vacuum samples taken at the territory boundary or within noants land often contained pairs of workers engaged in a fight (arrows), suggesting that workers from adjacent colonies occasionally wander into “enemy territory.” The focal nest was at the origin of this map, which also shows the inner and outer territory boundaries and the number of scouts captured at each sample location. Mapping used polar coordinates. High quality figures are available online.

**Boundaries and No-ants' land:** Vacuum samples taken between the inner and outer territorial points often netted few fire ants or none at all. Each territory was thus separated from its neighbors by a zone with very low forager density, a kind of “no-ants' land.” Bait tubes placed in this zone often remained unoccupied by fire ants, or were occupied by other species, such as *Brachymyrmex musculus, Pheidole floridanus* or *P. moerens*. Vacuum samples in these zones often netted *Cyphomyrmex rimosus, P. moerens* or *Hypoponera opacior*, but no fire ants. Although this made the territories appear completely exclusive, with no intermingling of neighboring fire ant foragers at all, some targeted vacuum samples showed otherwise. For the third colony of the Miccosukee Greenway set, the area was not only vacuumsampled well within the territory, but also immediately inside the focal colony and neighboring territories. Fighting pairs of workers were seen in four of these 16 nearboundary samples (arrows, Figure 12), suggesting that a few workers from each colony regularly wandered into the territory of their neighbor (the no-ants' land was narrow for this colony). This observation has important implications for the manner in which territorial boundaries are formed, as discussed below.

### Forager size distribution

It has been shown that mean worker body size, as measured by headwidth across the eyes, increases with colony size (Tschinkel 1993). Much of this mean size increase is the result of an increase in the proportion of major workers as colonies grow. Thus, for every 10 m^2^ increase in territory area, the proportion of major workers (those with headwidths >0.7 mm) in the vacuum forager samples increased approximately 3%. Colonies of less than 20 m^2^ averaged about 10% majors, whereas colonies over 100 m^2^ had 40 to 55% majors.

It also seemed possible that these major workers might not be evenly distributed with respect to distance from the nest, but this was not the case. The percent of major workers in the vacuum samples was not related to either the distance from the nest or the fraction of the distance between the nest and the boundary. There is no evidence that major workers and minor workers occupy the territorial space differently.

**Figure 13.**
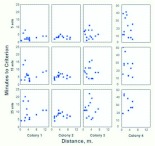
The time required for 5, 15 and 25 foragers to accumulate on baits at various distances from the nest. While it obviously took longer to accumulate more ants, this accumulation did not depend on distance from the nest, suggesting that the accumulated foragers did not emanate from the nest, but from nearby in the foraging tunnels. Note the different time scale for colony 4. High quality figures are available online.

**Where is the reservoir of foragers located?** Most ant colonies, including *S. invicta*, are central place foragers, returning foraged food to their colony. The recruits to food discoveries probably emanate from the central nest in most species, but in fire ants, this seemed less likely, because initial recruiting times seemed too short for recruits to have come from the central mound. In the spring of 1997, together with undergraduate student Bert Williams, the speed of early recruitment was tested as a function of distance from the nest. The boundaries of four colonies were mapped, and baits were placed at 1 to 2 m intervals on three radii emanating from the focal colony. The time it took for 5, 15 and 25 workers to appear on each bait was recorded. It was not surprising that it took significantly longer to accumulate more ants, with 5 ants appearing in a mean of 6.1 minutes, 15 ants in 10.7 and 25 in 14.0, a roughly linear relationship. Of greater interest was that none of these levels of accumulation depended on distance from the nest (Figure 13). Even with distances as great as 13 m, there was no relationship between distance and the time it took to accumulate a specified number of ants (multiple regression, F_1,55_= 1.144; p∼ 0.25; R^2^ = 0.78%). This is especially obvious when colony 4 is ignored. This colony gave highly erratic results, sometimes failing to recruit to baits at all.

### Running speed

Circular concrete barriers were constructed around mounds, forcing underground foraging traffic to cross the concrete, that allowed workers to be seen, counted and captured. Workers were timed as they crossed these 8 to 10 cm stretches. Running speed averaged 1.96 cm/sec (SD 0.64, n=15). At approximately 2 cm/sec, a worker could travel 1 m of foraging tunnel every 50 seconds. Heavier traffic slowed this speed because of interference, but 1 m/min is probably an acceptable figure. A worker running without pause after departing from a bait 5 m from the mound would thus reach the mound in 5 min. Allowing some time for recruitment, and a 5 min outward trip, mound recruits can be expected to make their first appearance at 5 m from the mound in 10 to 15 min. Longer distances would take proportionally longer.

From these results, it seemed more likely that at least some of the recruits were “stationed” out in the territories, perhaps in the underground foraging tunnels that underlie each territory, and that these recruits travel relatively short distances to arrive at the food. By blocking access to the nest from half the territory, but not the other half, the following experiments tested whether recruits were drawn from the field, from the nest or both.

### Blocking colony access

For the first colony, blocking was achieved by cutting a narrow trench across mapped territory, filling this trench with quick-setting cement and adding an aluminum flashing wall on top. The trench passed just to one side of the mound. The territories of the second and third experimental colonies were divided in a less laborious manner by hammering galvanized sheet metal flashing approximately 15 cm into the ground. Six to eight small boards containing strips of Spam were placed at predetermined locations in each half of the divided territory. Initially, workers on the baits were counted, but when there were too many to count, they were collected into boxes with fluon barriers and weighed. Most experiments ran from 1.5 to 2 hours, during which time no ants were observed climbing the barrier.

The effectiveness of blocking access to the colony was tested by adding a casamino acid/sugar solution dyed with 1% rhodamine B to the Spam baits on the access-blocked side and undyed solution to the access-allowed side. Presence of the dye in workers at the baits was checked by crushing workers on glassine paper and inspecting for orange fluorescence under ultraviolet light. The fact that, in two replicates, the dye was consistently absent from 293 and 607 workers taken from the mound after two hours of baiting showed that the barrier truly blocked off the mound from the territory. By contrast, 23 and 42% of the 151 and 156 workers taken from baits on the access-blocked side contained the dye.

At the end of a run, the barrier was removed (in the first run, only the-third of the barrier near the colony was removed), allowing the colony to reestablish its foraging tunnel connections with the previously blocked part of its territory. Two to seven days later, the barrier was reinstalled, this time passing around the previously unblocked side of the mound, so that the half of the territory that was open in the first run was now blocked, and vice versa. As done previously, Spam baits were placed at the same locations, and sampling proceeded as in the first run.

**Figure 14.**
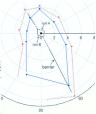
Map showing how a territory was cut in half with a barrier so that one half of the territory was accessible from the nest, but the other was not (run A). A day or more later, this was modified so that accessibility of the two halves was reversed (run B). The barrier shown by solid lines was either left in place for both runs, or was reinstalled for the second run. The barrier shown as dotted was removed between runs to allow the colony to re-establish underground foraging tunnel connections. The nest is at the origin of the polar coordinates and distances are shown in meters. High quality figures are available online.

Data consisted of the cumulative number of ants recruited to each bait as a function of time. In the first replicate, this was determined by counting until there were too many ants on the baits to count. Thereupon, at intervals, the ants were tapped into a fluoned sandwich box and weighed. Counts were calculated from the mean worker weight derived from a counted sample. In the second and third experiment, the bait boards were photographed with a digital camera, and the ants counted from these images. As done previously, when the baits were covered with workers, the workers were tapped into a box, and a second photograph taken. The difference in the counts of these two photographs yielded the number of workers tapped into the box. These counts were cumulated over the course of the experiment (Figure 14).

**Figure 15.**
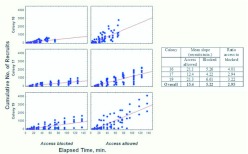
The rate of forager accumulation on baits when access to the nest was allowed or blocked. Recruitment to access-allowed baits was about three times as fast as to the access-blocked baits, suggesting that the majority of recruits normally come from the nest, not the field. High quality figures are available online.

**Estimating recruits waiting in the nest:** Although the following calculations should probably be considered rather tentative, a comparison of the cumulative buildup of recruits on baits with access to the colony allowed and blocked gives an estimate of the allocation of recruitable workers to the nest and the territory. Figure 14 shows this comparison for all six runs on the three experimental colonies. When access was allowed, recruits accumulated an average of about 3 times as fast as when it is blocked (Figure 15), and the final cumulative total number of recruits on the access-allowed baits is about 3 times that on the blocked (1285 vs. 438; ANOVA on log final cumulative totals: treatment effect — F1,77 = 32.4; p< 0.00001; colony effect — n.s.). This suggests that about one-third of the recruitable workers wait in the field, whereas two-thirds wait in the nest. Vacuum vs. mark-recapture estimation (see above) showed that this recruitable population composed about 55% of the total foraging force. About one-third of these, perhaps 18 to 20% of the total, seem to wait in the field, away from the nest. It seems likely that they wait in the foraging tunnels.

However, all territory-division experiments were done on large colonies. It seemed possible that the nest vs. field distribution of recruitable workers might vary with colony size. In the mark-recapture studies (Figures 2–8), the proportion of the foragers that were recaptured in the nest during excavation was estimated, rather than in the field during markrecapture operations. Figure 15 shows the fraction of the forager force that was in the field, rather than in the nest, at the time of nest excavation and census. There was a correlation between the proportion of a colony that foraged, and the proportion of these foragers that were in the territory rather than in the nest. Thus, small colonies, which typically field a higher proportion of their work force as foragers, also post a higher proportion of these in the territory. Reversing the logic, larger colonies with lower proportions of foragers retain a greater fraction of these in the nest. This pattern is in keeping with the increase of the reserve force in larger colonies (Tschinkel 2006).

**Figure 16.**
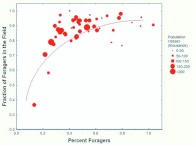
As the fraction of a colony that can be recruited to food increases, the proportion of these deployed to the field also increases. Thus, small colonies, which have a higher proportion of foragers, post a greater proportion of these in the territory. Symbols are coded for colony size, showing that small colonies generally field larger proportions. High quality figures are available online.

**The role of underground foraging tunnels:** Foraging traffic flows to and from the foraging territory in underground tunnels that were first described by Markin et al. (1975), who mapped tunnels by casting them with molten lead. Casting was begun by trenching around the nest to expose the severed foraging tunnels, then working outward, casting with molten zinc (Tschinkel 2010) 50 to 150 cm of tunnel at a time. The cast segments were exposed but left in place until the entire system had been cast, and a digital photomosaic had been made (Figure 16). Thereafter, the segments were labeled and returned to the laboratory for further analysis.

**Figure 17.**
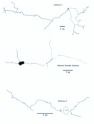
Zinc casts of the foraging tunnel system of three fire ant colonies, reconstructed in their natural configuration. The black or open circle indicates the nest mound. Tunnels were mostly 1 to 3 cm below the ground surface, with openings to the surface at intervals. Note the difference in scales. The longest tunnel in this case was over 15 m. The tunnel images have been digitally widened because their length-to-width ratio would otherwise make them difficult to see. High quality figures are available online.

Tunnel morphology ranged from simple, round cross-sections with smooth walls to wide ribbons with many smaller intersecting passages (Figure 17). All casts had openings to the surface at intervals of 50 to 100 cm, recognizable by a short, vertical shaft that sometimes allowed the molten zinc to puddle on the surface. Some shafts did not appear to open to the surface, but were probably readily opened on demand.

**Figure 18.**
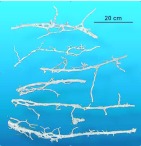
Most underground foraging tunnels are simple, cylindrical, occasionally-branching structures. The tunnel casts shown here were unusual in that they were much more complex, with diverging and converging, interlacing and frequently branched structure. The reason for this is not clear, but may have been related to the one-sided traffic in this territorially constrained colony. High quality figures are available online.

Tunnel dimensions generally decreased with distance from the nest (Figure 18), although the roughness of the cast made these measurements rather variable. Such a decrease might be expected if traffic divides into branches and some moves to the ground surface, much as the tributaries of a river system or the vessels of a circulatory system possess channels in proportion to the flow they carry. However, whereas the sum of the cross-sectional areas of the vessels or rivers is expected to be constant at any branch order, this is unlikely to be true for the foraging trail system of fire ants, because traffic is lost to the tunnels when the ants exit to the ground surface.

Figure 18 shows that tunnels decrease to what is possibly a lower limit, and this decrease occurs at a rate inversely proportional to the tunnel length. Because long tunnels service a larger area, they probably carry more traffic, funneling workers off to the surface throughout their entire length, and thus slowing the reduction of tunnel size.

**Figure 19.**
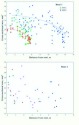
The cross-sectional area of tunnels of nests 1 (top) and 3 (bottom) in relation to distance from the nest. Each color symbol is a different tunnel emanating from the nest or branching from a larger tunnel. In nest 1, tunnels are identified by their initial compass heading as they originate from the nest. The tunnels are of greatly different lengths but decrease similarly in size, so that the decline of size is slower for the longer tunnel. Data points include secondary and tertiary branches. High quality figures are available online.

The carbonized bodies of ants were visible in the surface of the casts, and considering the wetness and low density of the ants in comparison with molten zinc, it seems likely that few ants would be incorporated into the body of the zinc cast, but would rather be pressed against the walls of the tunnels (Figure 19).

**Figure 20.**
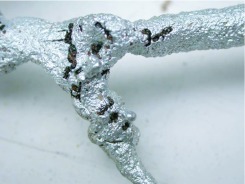
The carbonized bodies of foragers were visible in the tunnel casts, and could be counted. High quality figures are available online.

The entire length of tunnels was marked off into 5 cm segments, and the number of ants in each segment counted. Segments that contained an entrance contained significantly more ants than adjacent segments or more distant segments (ANOVA; Figure 20), suggesting that the carbonized ants were recruits waiting near an exit to be recruited by scouts.

The Alumni Center cast (Figure 17) was not analyzed for worker corpses because the extreme complexity of the tunnels made it difficult to identify openings to the surface from other kinds of intersecting passages. Why these tunnels were so complex is unknown.

**Are foragers faithful to a particular route?** It seemed possible that recruits would return repeatedly to a particular tunnel to await their next job. Such route faithfulness has been described in a number of different ant species (Hölldobler and Wilson 1990). To test this, a large number of workers were captured at two well-separated points within a territory. Workers (n= 2432) captured at one point were marked with orange fluorescent ink and those at the other (n= 2090) with yellow ink, and released at their points of capture. If marked workers tend to return to the same foraging tunnel, they will not mix randomly with the general forager population, and there should be a much lower percentage of marked workers captured on radii other than the one on which the workers were captured (where the capture rate should be high). On the other hand, if they lack faithfulness to their original tunnel, they should appear at similar mark rates on all radii.

**Figure 21.**
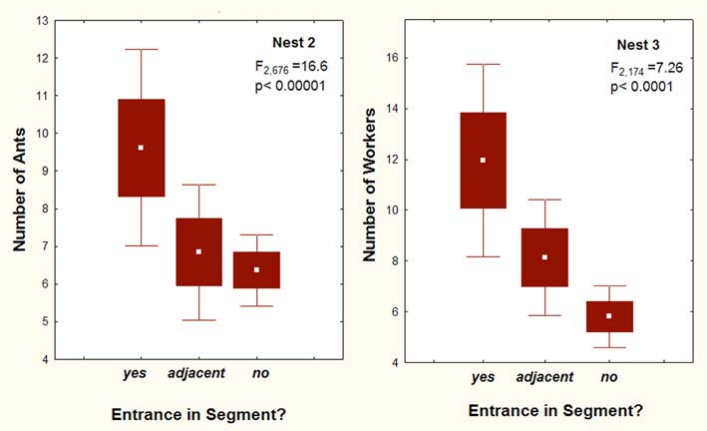
The number of ants in cast segments containing an entrance to the surface, adjacent to such a segment, or more distant. Ants were more frequent in segments with entrances, suggesting that these ants were recruits waiting to be recruited by scouts. In a third cast (not shown), the pattern was not as strong. High quality figures are available online.

However, the former was not the case. Figure 21 shows the percentage of each color recaptured at all bait points on the day after marking and release. Assuming that the baits on each radius draw from different foraging tunnels, an analysis of variance of the capture rates on radii estimates the degree of homogeneity of marked worker redistribution. There were a few significant differences in the mark rates among radii, both on day 1 and subsequent days (not shown). How these arose, and what they mean are questions for future experiments. The point here is that marked workers appear at variable rates on all radii, not just the one on which they were captured. In other words, they do not return only to the tunnel from which they were captured. The higher rates of recapture on the original radius are probably because some workers that did not return to the nest after being released, but simply returned to their stations in the same tunnel from which they had come. It is clear is that workers that return to the nest after marking are likely to choose another tunnel for return to the field. How they make this choice is not known.

**Figure 22.**
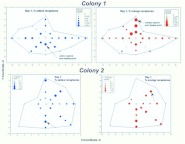
One day after foragers on baits were marked and released, they were recaptured throughout the territory, showing that they were not faithful to any particular route. Foragers were captured and released at two different points, and were marked yellow at one and orange at the other. The nest is at the origin, and the capture-and-release points are indicated by arrows. High quality figures are available online.

A more critical test of tunnel faithfulness was performed as follows. A large number of workers were again captured on baits at two well-separated points within the territory. Half of the workers at each capture point were painted orange (n= 3900) and half yellow (n=4000). Yellow workers were released at the same capture point and orange at the opposite capture point. In the absence of route fidelity, the proportion of colors in recapture samples should be about 1:1. Figure 22 shows that most of the recapture samples were in the 40 to 60% orange, with a few higher and a few lower. The distribution of ratios was not significantly different than a normal distribution with a mean of 0.53 (χ^2^ = 2.99, df = 2 (adjusted), p = 0.22).

**Figure 23.**
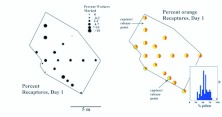
A more critical test of the lack of route-faithfulness. Workers were captured at two points within the territory (arrows), half were marked orange and half yellow. Yellow foragers were released at the capture point, and orange at the opposite point. If workers disperse randomly over the territory, then the ratio of orange to yellow when recaptured throughout the territory a day later should be about 1:1. This condition is met at most recapture points. The histogram shows the actual distribution of frequencies, which are summarized as classes in the pie diagrams in the right figure. High quality figures are available online.

**Territory in relation to foraging conditions:** By late May, 2006, when the project was started at the Miccosukee Greenway, the Tallahassee area had been without rain for almost 4 weeks, and soil and surface conditions were extremely dry. Bait tubes laid out to determine territory boundaries often failed to attract any ants at all, and there appeared to be very large unoccupied spaces (no-ants' land) between neighboring territories (Figure 23A). This zone was up to 6 m wide. Vacuum sampling confirmed that whereas foragers were active on the surface within the mapped territory boundaries, samples within the no-ants' land were usually devoid of fire ants (or any other ants).

This picture changed greatly after a heavy rain on May 25. By the next sampling day, June 1, much of the no-ants' land had been occupied either by the focal colony or by its neighbors (Figure 23B).

Although these observations were fortuitous and unreplicated, they suggest that under conditions unfavorable to foraging, or perhaps even to being above ground, the forager force does not merely thin out but actually retracts toward the nest, leaving the more distant zones of its territory unoccupied. In support of this conclusion, no subsequent territories had large no-ants' lands, and of course, all were determined after the drought period had ended.

### General Discussion Allocation of labor to foraging

Because of the multiple interrelationships among the colony size-area-forager data, several interpretations are possible. I have chosen an interpretation that integrates what is known about the annual and life cycle of the fire ant as described by Tschinkel (1993; 2006) with the findings I presented here. The patterns seem to be driven by major life history and demographic attributes as follows: (1) as a “weedy” species (Tschinkel 2006), *S. invicta* must produce a very large number of sexuals, but cannot do so while maintaining worker production above replacement rate, causing colony size to decline during the spring sexual production period; (2) worker lifespan is relatively short (50–150 days), resulting in an annual turnover of over 300% (Tschinkel 2006); (3) the large seasonal variation in worker production causes the agestructure of the worker population to vary greatly seasonally; (4) foragers defend and hold territory and are drawn, with only limited flexibility, from the oldest age group of workers; (5) foragers are depleted disproportionally in colonies producing sexuals in the spring, causing forager density to decline with colony size; (6) colonies producing few or no sexuals build up their worker populations throughout the warm season, fielding a large proportion of their older workers as foragers in the fall; (7) over the annual cycle, small colonies therefore gain territory at the expense of large ones, creating a mechanism that ratchets small colonies upward in both territory and colony size, and large colonies downward. (8) Territory loss and gain are buffered against moderate changes of forager numbers, and are probably rather slow, assuring that forager losses are not immediately or proportionally represented as territory losses.

This description applies to an average colony. The fact that colonies of the same size can differ by 4-fold or more in area hints at other factors that help determine the realized territory area. Chief among these is the territorial pressure from neighbors, a conclusion that is supported by the large variation in forager density associated with territory area (rather than colony size), and the small amount associated with percentage foraging. Moreover, colonies do not all grow to the same size. In natural populations, many go through their entire lives at a small or medium size, while others grow large and still others vary or grow smaller (Tschinkel 2006). Clearly, the fate of any particular colony depends on many factors, some described here, but many factors are poorly understood or unknown.

There are few studies for comparison, and none in which the scaling of foraging effort to colony size through the life cycle was determined. Few patterns across species have been documented. Very few studies of censused colonies support the oft-repeated claim that only a small proportion of an ant colony forages, but even this is not consistent across species. For example, in colonies of *Pachycondyla caffraria* (Agbogba and Howse 1992) and *Odontomachus brunneus* (LM Hart and WR Tschinkel, unpublished data) most of the ants forage. There is no consistent pattern with respect to colony size either — in both the large colonies of *Pogonomyrmex owyhee* and the small colonies of *Pachycondyla apicalis* 7 to 15% forage. Moreover, in *Formica polyctena*, the forager population is isometric with colony size (Kruk-DeBruin et al. 1977). The phylogenetic position of the species does not help either, for both species of *Pachycondyla* and *Odontomachus* belong to the “primitive” subfamily Ponerinae. More recently, C. Kwapich and Tschinkel (unpublished data) found that, whereas the proportion of Florida harvester ant colonies that forages is unrelated to colony size, it declines with season. In the early summer, about 35% of the colony foraged, but by October this declined to about 10%. My interpretation (see above) would suggest that this should be true in the spring in *S. invicta*, but the data show no such pattern because they were collected over too short a period to detect a decline. However, the harvester ant data support the seasonal loss of foragers that are part of my interpretation of the *S. invicta* data. At this time, the data are too few and diverse to discern any patterns in the allocation of labor to foraging, and the factors selecting the level of such allocation are unknown.

Even so, it seems obvious that how much labor a colony allocates to various types of tasks must be under strong natural selection. Effective foraging pays dividends in colony fitness, as has been shown by Cole et al. (2008). Colonies that forage longer bring in more food, fueling colony growth and sexual production. Although the basis in this case was genetic diversity, it seems likely that any factors affecting fire ant foraging will also affect colony fitness. Other than Cole et al. (2008), no such factors have been identified.

The importance of labor allocation is made even more obvious by the fact that neither division of labor by age or by body size may be very flexible, committing the colony to a rather narrow allocation pattern based on the mix of worker ages and body sizes present at the moment. For, example, in *S. invicta* large workers do not care for brood under any circumstance (Porter and Tschinkel 1985), and older minor workers decline strongly in the efficiency with which they perform tasks characteristic of younger workers (Cassill and Tschinkel 1999). For example, it is unlikely that a colony can draw effectively on large workers to carry out the tasks of small workers that were accidentally lost. Similarly, foragers are unlikely to return to the nest to carry out brood care, especially in species with such extensive territories as fire ants. Reversal of the life trajectory of a worker is probably possible only to a limited extent. It is this fact that probably drives the demographic seasonal swings that I interpreted (see above) as causing the seasonal variation in forager populations as well as the relationship between foragers and territory area.

### Spatial patterns

Recruitable foragers are a reserve labor force waiting to be called to action by scouts. About two-thirds of these reserves wait in the nest, probably in its periphery near the ground surface and the roots of the foraging tunnels. Another third is stationed in a dispersed manner in the foraging tunnels, where they can respond rapidly to recruitment by scouts. Most of the area within a territory is a meter or less from the opening into a foraging tunnel (Markin et al. 1975), greatly reducing the travel distance and time for the recruits in the tunnels. In foraging tunnel casts, workers were more abundant near entrances/exits, suggesting that this is where recruits wait, this reduces the response time even more. The system seems geared for rapid response to food bonanzas, items that are too large for a single, or a few workers to handle.

When even the local recruits from the tunnels are not sufficient, the wave of recruitment echoes back to the nest and the much larger recruit force waiting there is called into action. Whether activation of nest recruits results from scouts returning all the way to the nest via foraging tunnels, or from the secondary actions of recruits is unknown. Of course, it could be both, but the fact that rewarded workers can themselves recruit more workers (Tschinkel 2006) suggests a kind of selfcatalytic mechanism for spreading the activation. In laboratory experiments, fire ant foraging is strongly regulated by the concentration of trail pheromone (Wilson 1965), but whether trail pheromone also plays this role in the foraging tunnels is unknown. The tunnel system branches repeatedly, and the recruits would have to be guided into the proper branch. The dynamics of this pheromone in the tunnels would probably be very different than on the surface. It is also interesting how a scout returning to the tunnel knows which direction is nestward. There is no evidence that fire ant pheromone trails, or any other ant pheromone trails for that matter, are in themselves polarized. In Pharoah's ant, the geometry of the trail bifurcations cues foragers as to the nestward direction (Jackson et al. 2004). Inspection of the tunnel system in Figure 16 suggests that tunnel geometry is not likely to be a directional cue in fire ants.

Once the recruitment event has played itself out, it seems that the recruits do not necessarily return to their original stations, as foragers marked at one location within the territory can be recruited at any other location a day later. This is in contrast to several other species in which workers exhibit various levels of “Ortstreu” (site fidelity) returning along the same route to the same site for extended periods. Hölldobler and Wilson (1990) cite more than 10 examples. More recent additions include *Formica obscuripes* (McIver and Loomis 1993) and *Acromyrmex niger* (Sousa-Souto et al. 2001).

It is interesting to speculate on the origin of the foraging tunnel system of *S. invicta*, first described by Markin et al. (1975). In certain ways it resembles the subsurface tunnel systems of the army ant *Dorylus (Dichthadia) laevigatus* (Berghoff et al. 2002) and probably other hypogaeic army ants. *D. laevigatus* maintains a stable system of underground tunnels, extending tunnels to rich food sources and enlarging them when recruiting larger workers. As with fire ants, most of the distance to food is covered underground, with relatively short raiding columns above ground. Of course, army ants are phylogenetically distant from fire ants, but the closely related thief ants (*Solenopsis* spp. formerly the subgenus *Diplorhoptrum*) are also subterranean foragers that maintain a widely dispersed 3-dimensional tunnel system connecting widely dispersed chambers (unpublished observations). Bringing the dispersed chambers of these thief ants to a central location while leaving the tunnel system intact would probably create something akin to a fire ant colony with its radiating foraging tunnels. This suggests a possible evolutionary pathway, or at least a kind of “ground plan” for the genus *Solenopsis*.

### Transport of food

When a large food item is discovered somewhere in the territory, a wave of recruitment clearly flows all the way back to the nest, but it is unknown whether individual scouts make this journey or a chain of recruitment carries the message back to the colony. Similarly, it is not known whether each load-carrying worker, whether bearing fluid in the crop or solids in the mandibles, carries this food all the way back to the colony or gives it up to a chain of transfer, like a bucket brigade (the direct transfer from worker to worker). The behaviors making such transfer possible are certainly present in fire ants as workers with fuller crops readily share fluid with workers with less full crops (Cassill and Tschinkel 1999). Similarly, dried pieces of insect prey are cached in the mound by fire ants, ready to be redistributed (Gayahan and Tschinkel 2008). Whether either of these transfers occurs in the foraging tunnels is unknown, but both caching and bucket brigades occur in leafcutter ants (Hubbell et al. 1980; Hart et al. 2001). Simulating bucket brigades suggested that the process is ergonomically efficient (Anderson et al. 2002), but leaf caching incurred the cost of mismatching the worker to the load when leaf pieces were picked up again (Hart et al. 2001). Such phenomena remain to be tested in fire ants. Another study of interest might be the traffic dynamics within the foraging tunnels, much like the study of leaf cutter ants on foraging trails (Burd et al. 2002; Dussutour et al. 2004).

### Boundaries and no-ants' land

A model based on outward pressure by territorial workers against neighbors, like an expanding gas, reasonably accounts for both the size and shape of fire ant territories (Adams 2003), but the processes that actually form the boundaries and the no-ants' land are not clear. Observations show that ants from neighboring territories occasionally wander across the no-ants' land, and fights occasionally occur there (personal observations). The most likely reason why the ant density in this boundary zone is low is that when neighboring foragers meet there, they either fight, or more frequently, rush back in the direction of their own territory, but this mechanism has not yet been tested. Territory could be more a product of avoidance than fighting. Such avoidance behavior at boundaries has been observed in *P. badius* by Harrison and Gentry (1981).

Adams (1990) showed that the likelihood that arboreal ants would gain or lose territory with respect to their neighbors depended on asymmetries in the strength of neighboring colonies. Arboreal species recruit to points of contact in their arboreal realm, a situation rather different from that of *S. invicta* where contact with neighbors is continuous around the entire perimeter of their territory. Hölldobler (1979) found similar recruitment to territorial intrusions in the arboreal *Oecophylla longinoda*, along with the existence of a “no-ants land” where adjacent territories met. Whether fire ants recruit to territorial intrusions is not known, although Adams (2003) was able to create battles by baiting them at the territory boundaries.

### The worker life cycle and foraging

It is practically universal among ants that as workers age, they move away from the brood area and gradually shift from brood care to general nest duties to foraging. This shift takes place in space as well as time, with the taskshifting workers moving upward in deep vertical nests (e.g. *Pogonomyrmex badius*, Tschinkel 1999) or outward in horizontally-organized ones (Sendova-Franks and Franks 1995). In fire ants, this spatial, behavioral and temporal transition takes place on a grand scale. As workers age, they move from the core of the nest where the brood resides to the nest periphery where they act as recruitable reserve workers. It is likely that as they age still more, they move outward to wait in the foraging tunnels, and it is certain that as they age still more, they leave the shelter of the tunnels to become scouts, roaming about the surface, exposed to myriad dangers. Scouting is the final phase of a worker's life and lasts only about two weeks, much as in *Pogonomyrmex owyhee* (Porter and Jorgenson 1981), terminating when the worker dies. The entire spatial pattern is driven by the outward movement of aging workers. Whether this movement occurs in discrete stages (nest recruits, tunnel recruits, scouts) or whether it is gradual and continuous is not known. If continual, then one might expect that even in the foraging tunnels, older workers would be in most distant parts of the tunnel system. The lack of route fidelity makes such rigidity unlikely.

In *F. polyctena*, foragers were also largely “stationed” in the field so that it required two days of trapping returning workers at the nest to capture the entire forager force (Kruk-DeBruin et al. 1977). Marking this forager population showed that even when in the nest, they were located only in the uppermost layers. It seems likely, though not yet established, that the recruitable workers of *S. invicta* also wait in the upper and peripheral regions of the nest. In keeping with this, marked workers were more abundant in the upper strata of my stratified excavations.

**Figure 24.**
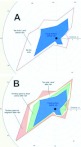
(A) Territory occupied by focal colony after a long drought, showing the large no-ants' land between occupied territories. (B) Within a few days after a heavy rain, much of the unoccupied space (no-ants' land) had been occupied by either the focal colony or its neighbors, and only a narrow sparsely-occupied zone remained. High quality figures are available online.
